# Effectiveness of work-related medical rehabilitation in cancer patients: study protocol of a cluster-randomized multicenter trial

**DOI:** 10.1186/s12885-016-2563-z

**Published:** 2016-07-27

**Authors:** Julian Wienert, Betje Schwarz, Matthias Bethge

**Affiliations:** Institute for Social Medicine and Epidemiology, University of Lübeck, Ratzeburger Allee 160, 23562 Lübeck, Germany

**Keywords:** Cancer, Medical rehabilitation, Work-related medical rehabilitation, Work-related therapies, Return to work, Inclusion, Cluster-randomized trial, Study protocol

## Abstract

**Background:**

Work is a central resource for cancer survivors as it not only provides income but also impacts health and quality of life. Additionally, work helps survivors to cope with the perceived critical life event. The German Pension Insurance provides medical rehabilitation for working-age patients with chronic diseases to improve and restore their work ability, and support returning to or staying at work, and thus tries to sustainably avoid health-related early retirement. Past research showed that conventional medical rehabilitation programs do not support returning to work sufficiently and that work-related medical rehabilitation programs report higher return-to-work rates across several health conditions, when compared to medical rehabilitation. Therefore, the current study protocol outlines an effectiveness study of such a program for cancer survivors.

**Methods:**

To evaluate the effectiveness of work-related medical rehabilitation in cancer patients we conduct a cluster-randomized multicenter trial. In total, 504 rehabilitation patients between 18 and 60 years with a Karnofsky Performance Status of ≥70 %, a preliminary positive social-medical prognosis of employability for at least 3 h/day within the next 6 months and an elevated risk of not returning to work will be recruited in four inpatient rehabilitation centers. Patients are randomized to the work-related medical rehabilitation program or the conventional medical rehabilitation program based on their week of arrival at each rehabilitation center. The work-related medical rehabilitation program comprises additional work-related diagnostics, multi-professional team meetings, an introductory session as well as work-related functional capacity training, work-related psychological groups, and social counseling. All additional components are aimed at the adjustment of the patients’ capacity in relation to their individual job demands. Role functioning defines the main study outcome and will be assessed with the EORTC-QLQ30. Secondary outcome measures are the remaining scales of the EORTC-QLQ30, fatigue, self-rated work ability, disease coping, participation in working life, realization of work-related goals and therapies during rehabilitation, and treatment satisfaction.

**Discussion:**

A positive evaluation of work-related medical rehabilitation in cancer patients is expected due to the promising findings on the effectiveness of such programs for patients with other health conditions. Results may support the dissemination of work-related medical rehabilitation programs in German cancer rehabilitation.

**Trial registration:**

German Clinical Trials Register DRKS00007770. Registered 13 May 2015.

**Electronic supplementary material:**

The online version of this article (doi:10.1186/s12885-016-2563-z) contains supplementary material, which is available to authorized users.

## Background

With approximately 14.1 million new cases in 2014, cancer is one of the leading causes of morbidity worldwide [1]. Europe alone accounts for 24.4 % (approximately 3.44 million cases) of the worldwide incidence. The prostate (22.8 %), lung (15.9 %), and colorectum (13.2 %) are the most prominent cancer sites among men in Europe. Among women, the breast (28.8 %), colorectum (12.7 %), and lung (7.4 %) are the most frequently affected sites [1]. These numbers also mirror the current situation in Germany where 477,300 incident cases were reported for 2010 [2].

Due to better screening and treatment of cancer, survival rates in cancer patients have improved steadily over the last few years in most developed countries. Five-year survival rates for cancer improved for patients diagnosed between 2005 and 2009 in most developed countries when compared to 5-year survival rates between 1995 and 1999 (e.g. Germany: 48.7 to 64.6 % for colon cancer, 51.9 to 62.1 % for rectal cancer, 81.2 to 85.3 % for breast cancer) [3]. As a result, the life situation of cancer survivors and their participation in different life domains received increasing attention from patients, clinicians, and researchers [4]. One of these important life domains is work. Work not only provides income to afford living, it also helps to structure time, enables social contacts, and develops skills and personality [5, 6]. The presence or absence of work has an impact on various facets of health and quality of life [7, 8]. Work also has a positive influence on coping with a disease (e.g. stroke [9, 10]; psychiatric disorders [11]; multiple sclerosis [12]). For cancer survivors, returning to work after cancer diagnosis and subsequent treatment may support their ability to cope with this critical life event and represents a major step in restoring normality [13–16].

Although up to 75 % of cancer survivors previously working in a job return to it after treatment [17], returning to work is often easier said than done. Cancer survivors are 1.4 times more likely to be unemployed compared to healthy controls [18]. They also have an increased risk of early retirement and are less likely to be re-employed [18, 19]. Reviews showed that a multitude of parameters influence the process of returning to work in cancer survivors, including health, well-being and symptoms, and different domains of functioning, as well as self-rated work ability and work-related factors such as job demands and the working environment [18–22]. In line with general return-to-work (RTW) research [23], RTW in cancer survivors can therefore be described as a multifaceted bio-psycho-socially moderated process. Consequently, comprehensive multidisciplinary rehabilitation strategies are favored for supporting RTW. A Cochrane review by de Boer et al. [19] confirmed that only diverse, multidisciplinary interventions show moderate evidence of higher RTW rates for cancer survivors when compared to usual care (for a comprehensive review across several health conditions, please see Hoefsmit et al. [24]). Established programs, however, are not necessarily multidisciplinary and often lack a specific focus on RTW [25]. In Germany, the German Pension Insurance (GPI) provides medical rehabilitation (MR) for working-age patients with chronic diseases to improve and restore their work ability, and support returning to or staying at work, and thus tries to sustainably avoid health-related early retirement. In Germany, about one fifth of all rehabilitation measures are provided due to cancer [26]. However, though improvement and restoration of work ability are the primary aims of German MR programs, conventional MR does not seem to address work-related issues sufficiently [27, 28]. The evidence considering the effects of these programs on work-related outcome measures in cancer patients is mixed at best. Most studies that are available did not use a control group and focused only on generic health-related outcome measures. These studies showed that MR can increase health-related quality of life, physical functioning, and general mental well-being [29–32], and decrease anxiety and depression [33]. Several studies, however, showed that, in particular, persons with a poor self-rated RTW prognosis did not benefit from conventional MR [34, 35]. Moreover, Weis and colleagues [32], who investigated the work-related impact of inpatient rehabilitation in female breast cancer patients in a controlled study, found no sustainable effects on health- or work-related outcomes [36]. Obviously, conventional MR as practiced in Germany is not a sufficient treatment to address the multifaceted factors of RTW in cancer survivors.

As a result, rehabilitation programs with a stronger focus on work, work ability, and RTW have been developed in recent years. By addressing the individual job demands and necessary skills to meet them, these programs aim to increase the chances of a sustainable participation in the labor market, especially for patients with more severe restrictions of their work ability (e.g. long or repeated periods of sick leave, unemployment, poor self-rated RTW prognosis). To provide a framework for these diverse programs and activities, the GPI published guidelines for the implementation of work-related medical rehabilitation (WMR) [37]. These guidelines define the target group, describe specific work-related diagnostic measures, and explain the core therapeutic modules that complement WMR programs as compared to conventional MR. The definition of the target group, i.e. patients on long-term sick leave and patients with a poor self-rated RTW prognosis, also includes recommendations for the identification of these patients by naming several screening instruments that assess an elevated risk of permanent work disability. The diagnostic process in WMR is described as demand-oriented, meaning that it should assess physical and psycho-social work ability against the background of the individual work-related demands. This also includes the identification of individual and environmental risk factors and resources that might be related to coping with these demands. Additionally, a functional capacity evaluation should be part of the diagnostic procedure [38, 39]. Finally, the core therapeutic modules of WMR as described by the guidelines are a) work-related functional capacity training, b) work-related psychological groups, and c) intensified social counseling [37].

Randomized controlled trials in patients with musculoskeletal [40, 41], cardiac [42], neurological [43], and psychosomatic disorders [44, 45] showed that patients who received WMR reported significantly higher RTW rates than patients who received a conventional MR program. Though WMR is well established for several disorders, there are currently only a few WMR programs for cancer patients. One non-randomized clinical trial in cancer patients showed no differences in RTW rates, work-related stress, and job satisfaction between a WMR group and a conventional MR group [46]. A major methodological shortcoming of this study was the imbalance of both groups regarding several baseline measures. Moreover, the description of the program indicates that it was not a comparably comprehensive strategy as described in the current guidelines for WMR. Therefore, we designed a study to determine the effects of well-implemented WMR programs on work-related and quality-of-life outcomes in cancer survivors.

## Methods

### Design

The study is designed as a cluster-randomized multicenter trial which is realized in four German rehabilitation centers: the Klinik Bavaria in Freyung, the Paracelsus-Klinik am See in Bad Gandersheim, the MediClin Rose Klinik Horn-Bad Meinberg, and the Röpersbergklinik in Ratzeburg. The control group receives conventional MR as prescribed by the German Pension Insurance, whereas the intervention group receives a WMR program. Patients are randomized in clusters. These clusters are defined by a shared start of the rehabilitation program. Randomization is stratified by centers.

### Recruitment

Patients are informed about the study by a study nurse or a physician in charge and have to provide written informed consent prior to study participation. Participants can withdraw their consent at any time without any consequences. No modifications to the intervention are planned. All rehabilitation centers were provided with a process description for recruitment prior to the study start and follow the same procedure.

When giving informed consent to participate in the study, the name and address will be listed by the rehabilitation center and a study number will be assigned. This study number will be entered in a field on each questionnaire. The study number will be also added to the medical discharge letter while all information on name and address will be removed to ensure that research data and confidential data cannot be linked. The study list with personal data and the study number will be destructed at the end of the study (12/31/2017) by each rehabilitation center to ensure that names and addresses of study participants are no longer available.

The researchers receive a copy of this study list only for mailing the questionnaires 3 and 12 months after discharge. Use of these data is not allowed for any other purpose but sending out the questionnaires. The study list will be secured with a password, stored separately from the collected research data, and erased after sending out the last questionnaires. The time schedule of enrolment and interventions is displayed in Table 1.

**Table 1:** Time schedule of enrolment and interventions

	Study period						
	Enrolment	Allocation	Post-allocation				Close-out
Time point	Pre-admission		Admission	Discharge	3-month follow-up	12-month follow-up	12-month follow-up
Enrolment:							
Eligibility screen	X						
Informed consent	X						
Allocation		X					
Interventions:							
Work-related medical rehabilitation							X
Conventional medical rehabilitation							X

### Treatment

#### Control

Participants in the control group receive a conventional MR as provided by rehabilitation centers in Germany. Conventional MR is usually a 3-week program with an overall amount of 60–75 h of therapy [26]. This includes exercise therapy, physiotherapy, social counseling, occupational therapy, psychological seminars and counseling, and dieting. Participants in the control group do not receive intensified work-related therapies. However, social counseling as an obligatory part of conventional MR informs on social law and provides basic knowledge about the rights of persons with disabilities. This also includes work-related issues.

#### Intervention

Participants in the intervention group receive a WMR program. In addition to the conventional MR, the WMR includes additional work-related therapies as defined by the WMR guideline (see above) [37]. This guideline prescribes at least 11–25 h of additional work-related content for WMR patients [37]. The complete treatment dose of a WMR program contains up to 100 h of therapy.

The intervention content is guided by the WMR guidelines [37]. Further specifications of the diagnostic and therapeutic procedures are achieved in collaboration with the rehabilitation centers, based on an implementation phase before the start of the initial trial. This implementation phase is aimed at increasing the feasibility and fidelity of therapies across all participating rehabilitation centers. This phase is also used to check whether centers prescribe WMR therapies in addition to conventional MR therapies for the intervention groups and that control groups only receive conventional MR. The additional content of WMR and work-specific therapies as well as their minimum amount of time are described below (see Table 2 for a brief overview).

**Table 2:** Brief overview of the WMR program

Module	Time	Setting	Professions	Content
Additional work-related diagnostics	During admission to rehabilitation Total: at least 60 minutes Medical assessment: 15 minutes Psychological assessment: 15 minutes Test of functional capacity and performance: 30 minutes	Personal interaction with the patient	Physician Psychologist, psychotherapist Occupational therapist, physiotherapist	Assessment of work functioning and its restrictions related to body functions and structures as well as activities and participation Assessment of environmental and personal factors that impact work functioning positively as resources and skills or negatively as barriers Use of structured patient interviews and/or standardized assessment instruments as well as standardized tests of functional capacity evaluation, structured observations of non-standardized job tasks
Multi-professional team meetings	After admission During the course of rehabilitation Before discharge	N/A	All professions associated with the WMR program	Individual case conference for each patient After admission: results of the work-related diagnostics, joint development of a treatment plan During the course of rehabilitation: development of the patient, adjustment of the treatment plan if needed Before discharge: evaluation of rehabilitation outcomes, return-to-work prognosis, additional measures to support return to work if needed
Introductory session	After admission	Presentation in front of all WMR patients	Physician	Description of the aims of WMR, explanation of the program structure and each module, introduction of the rehabilitation team Inform, motivate and prepare the patient for the subsequent program Establish treatment collaboration
Work-related functional capacity training	At least 360 minutes	Training in small groups or personal training	Occupational therapist, physiotherapist	Complex and multidimensional tasks to simulate realistic work-related demands and situations Integrated ergonomic and cognitive training
Work-related psychological groups	At least 240 minutes	Seminars with small groups	Psychologist, psychotherapist	Seminars focusing on work-related stress and coping, work-related social competencies in communication, and planning the concrete return to work
Intensified social counseling	At least 90 minutes: 60 minutes seminars 30 minutes personal counseling	Seminars with small groups or personal counseling	Social worker	Clarification of the problematic work-related situations and perspectives, information and consultation on social law-related topics as well as measures and benefits to support return to work, establish contact with employer and request further measures and benefits from the social or health services agencies if needed

##### Additional work-related diagnostics

Additional work-related diagnostics are carried out by the physician in charge, a psychologist and an occupational therapist or physiotherapist directly after admission of the patients. The diagnostic procedures are aimed at a comprehensive assessment of work functioning. Work functioning is understood as the result of the interaction between the health problem on the one hand and work-related environmental and personal factors on the other. Restrictions in work functioning can be related to body functions and structures, activities, and participation [47, 48]. Each of the three involved professions makes its own specific contribution to the comprehensive assessment. The physician primarily focuses on body functions and structures (e.g. impairments). The psychological assessment, comprising interview techniques as well as standardized instruments, focuses on environmental (e.g. support of co-workers and supervisor) and personal (e.g. coping styles) factors that impact work functioning positively as resources and skills or negatively as barriers. Finally, the occupational therapist or physiotherapist assesses the ability to perform work activities by using standardized tests of functional capacity evaluation or structured observations of non-standardized job tasks [38, 39]. The complete diagnostic process takes at least 60 min. The medical and the psychological assessment take at least 15 min each. The assessment of work-related functional capacity takes at least 30 min. On the basis of the diagnostic findings, the multi-professional team jointly develops a comprehensive rehabilitation plan with a tailored treatment strategy for each patient.

##### Multi-professional team meetings

Multi-professional team meetings are carried out once a week by the WMR team, i.e. by the physician in charge, the psychologist, the social worker, and at least one occupational therapist and physiotherapist. The meetings provide a platform for mutual exchange about the single patients assigned to the WMR program (case conference). Besides facilitating and bundling communication about the patients, the meetings also aim to coordinate the single WMR elements provided by the different team members to achieve concerted action. The first meeting takes place directly after the aforementioned diagnostic procedures to jointly develop a rehabilitation plan and treatment strategy for each patient, focusing on the enhancement of work functioning. In the second meeting all team members report on the patients’ progress and current situation, assessed or observed during or alongside the different therapeutic interventions. If necessary, rehabilitation plans and treatment strategies are adjusted. In the last meeting the individual rehabilitation outcomes are evaluated. Based on this evaluation the final decision on return to work or prolonged sick leave is discussed within the team. Moreover, additional measures and benefits to support return to work are talked about; if needed, recommendations are given to the GPI. Each team meeting takes at least 30 min.

##### Introductory session

The introductory session informs all patients who participate in the WMR program about the structure, content, and aims of the program. Each therapist is introduced with their field of expertise so patients can identify the right person to address expertise-related questions. The session is organized as a group presentation, which is held by a physician at the beginning of the WMR program and takes a minimum of 30 min. The session is aimed at 1) getting patients familiar with the program right from the beginning of their treatment, 2) initiating a treatment collaboration between patients and therapists (i.e. a respectful and trustful relationship between patient and therapist) by providing a transparent structure, and 3) motivating patients to actively participate in their therapy by providing additional information about the potential benefits of the program.

##### Work-related functional capacity training

Work-related functional capacity training is carried out by an occupational therapist or a physiotherapist in group or personal sessions. Based on diagnostic results and following principles of graded activity (e.g. [49]), an individualized training plan is developed for each WMR patient to purposively increase work-related capacity and facilitate work-related performance. Graded activity comprises regular training with general exercises, strengthening exercises, and tailored exercises that imitate individually relevant physical tasks by making use of operant-conditioning behavioral principles with the aim of restoring functioning. The primary aim is not pain relief. However, participants learn that despite pain symptoms, exercise and physical activity are safe and recommended [49]. Following these principles, training plans not only contain single or one-dimensional motion tasks (e.g. latissimus pull-down exercise), but also complex and multidimensional tasks (e.g. lifting and carrying bricks, tightening bolts overhead, filling a goods shelf, patient transfer) to simulate realistic work-related demands and situations. Simultaneously, therapists train patients in ergonomic working by implementing ergonomics in the regular training (e.g. ergonomic corrections when lifting goods onto a shelf).

Since cognitive impairments (as a symptom, result, or side effect of the disease or its treatment) are often reported by cancer survivors, this module also comprises cognitive training sessions. Amongst others, computer-assisted training or group sessions under the guidance of a therapist are used to improve attention, concentration, memory, and logical thinking. Work-related functional capacity training requires a minimum of 360 min of therapy.

##### Work-related psychological groups

Work-related psychological groups are provided by a psychologist and have the purpose of supporting the concrete RTW process of each patient by focusing on personal and environmental factors. In the group sessions the patients are encouraged to reflect critically on their current job situations and past work-related behaviors and on how they might be able to adjust themselves or the situations accordingly. Therefore, the patients learn about stress and its influence on health and work ability and are taught about diverse coping strategies (e.g. active coping, seeking instrumental or emotional support, positive re-evaluation). Group work is used to deepen the newly acquired knowledge and to transfer it to individual conditions. Techniques for immediate stress reduction such as progressive muscle relaxation, autogenic training, and meditation are shown and practiced to support coping. Additionally, communication theories and techniques are presented to the patients focusing on interpersonal relationships and communication with colleagues and supervisors. Role plays that take up realistic or even personally experienced situations at the workplace can be used to put the theory into practice. As part of work-related psychological groups, the patients learn how to apply meta-cognitions on how to achieve their RTW aims in the best possible way. To do so, they have to develop and schedule concrete RTW plans for the time after discharge from rehabilitation, taking into consideration also potential problems and barriers, as well as strategies to tackle them. Questions to clarify are: “What?”, “When?”, “With whom?”, “How?”, and “What if?”. Plans are created by considering the RTW framework that is provided as the final result of social counseling. Work-related psychological groups cover at least 240 min of group therapy. Additional personal counseling can be provided at the request of the patient.

##### Intensified social counseling

Additional social counseling is carried out by a social worker in group and in individual sessions. It is aimed at the clarification of the problematic work-related situations and perspectives of WMR patients by providing them with extra information and advice on social law and services. The social worker develops the RTW framework jointly with the patient and identifies which additional measures and benefits are needed and available to achieve RTW in each specific case. Such measures and benefits cover a broad scale of possible instruments and range from modifications of the work environment (e.g. height-adjustable desks) through vocational retraining (e.g. achieving a new professional title), to wage substitutes. The social worker requests the individually indicated measures and benefits from the social or health services agencies (e.g. GPI, health insurance, integration office). If needed and wanted by the patients, the social worker also establishes contact with the employer to facilitate the transition from the rehabilitation center back to the workplace (e.g. by initiating graded RTW or modifications of the work environment). The group sessions take at least 60 min. The personal counseling takes at least 30 min.

### Participants

Cancer patients (ICD-10: C00-D48) aged 18 to 60 years are included in the study if they have a score of ≥70 % on the Karnofsky Performance Status Scale [50, 51] and a preliminary positive social-medical prognosis of employability for at least 3 h/day within the next 6 months as assessed by the rehabilitation physician in the initial examination after starting the rehabilitation program. Moreover, patients are only eligible to participate if they have an elevated risk of not returning to work. This risk is assessed by the Screening Instrument Work and Occupation (German: Screening-Instrument Beruf und Arbeit in der Rehabilitation, SIBAR) [52–54]. This screening consists of three subscales. The first subscale is a 9-item scale and assesses the risk of health-related early retirement on the basis of a weighted sum score of several risk indicators (e.g. sick leave duration and the patients’ belief of time to return to work). A score of more than 7 out of 19 possible points indicates an elevated risk of early retirement and thus a need for a WMR program. The second subscale is a 1-item score and assesses the amount of perceived job strain. The third scale is also a 1-item score and assesses the subjective need for work-related therapies during the rehabilitation program. If patients indicate a very stressful job situation or assume that work-related therapies will be very helpful, this is also taken as a marker of the need for a WMR program. In brief, patients are included if one of the three scales indicates a need for a WMR program. Previous studies estimated that this is the case for about half of the patients. Moreover, about a quarter of the patients has an elevated risk of early retirement based on the first scale of the SIBAR [52–54]. No exclusion criteria were defined.

### Randomization

The study is designed as a cluster-randomized multicenter trial. Patients are recruited from four inpatient rehabilitation centers. Patients of a rehabilitation center who start their rehabilitation in the same week represent a cluster. These patients jointly receive either the intervention or the control treatment. Cluster-randomization was chosen to avoid spillover effects between therapy groups. Each of the four rehabilitation centers receives its own randomization schedule. The randomization lists are created by the last author using computer-generated random numbers and blocks of 4. The administrative employees who assign the patients to their date of rehabilitation are blinded to the randomization schedule.

### Sample size calculation

Sample size calculation was done to detect a standardized mean difference (SMD) of SMD = 0.3 in the primary outcome with 80 % power and a two-sided alpha error of 5 %. Using a t-test, a sample size of 352 participants would be needed. The planned regression modeling of treatment effects, which includes the baseline measurement of the primary outcome as a covariate, needs a sample size that is reduced by the factor 1 - r^2^ [55]. The correlation r represents the correlation between baseline and follow-up measurement. The approximation of the sample size based on a t-test represents a conservative proxy, even when considering the design effects induced by the cluster-randomization or missing values. Based on expected sample attrition due to nonresponse to the 1-year follow-up questionnaire of 30 %, we aim to recruit at least 504 patients.

### Outcome measures

Primary and secondary outcomes are assessed by questionnaires at the beginning of the inpatient rehabilitation program, at discharge from the rehabilitation program, and 3 and 12 months after discharge. Questionnaires at the beginning and discharge of the program are delivered and collected by study nurses in the rehabilitation centers. These nurses are not blinded to the treatment allocation. Questionnaires at 3- and 12-month follow-up are sent by mail to the first author who is also not blinded to the treatment allocation. Table 3 shows all variables and their corresponding measurement point.

**Table 3:** Time schedule of assessments and instruments

	Admission	Discharge	3-month follow-up	12-month follow-up
Role functioning (EORTC QLQ-C30) [56]	X		X	X
Physical functioning (EORTC QLQ-C30) [56]	X	X	X	X
Emotional functioning (EORTC QLQ-C30) [56]	X	X	X	X
Social functioning (EORTC QLQ-C30) [56]	X		X	X
Pain (EORTC QLQ-C30) [56]	X	X	X	X
Global health (EORTC QLQ-C30) [56]	X	X	X	X
Fatigue (EORTC QLQ-FA13)	X	X	X	X
Coping with illness (FCQI) [60]	X	X	X	X
Implementation of work-related therapies [63]		X		
Consistency of work-related rehabilitation strategy [63]		X		
Benefit from work-related therapies [63]		X		
Work Ability Score [59]	X	X	X	X
Employment status	X		X	X
Time of return to work			X	X
Sick leave duration	X		X	X
Disability days during the last 3 months [61]	X		X	X
Patient satisfaction [64]		X		
Sociodemographic data	X			
Discrete choice experiment	X			
Screening Instrument Work and Occupation [67]	X			

*EORTC QLQ-30* 30-item quality-of-life questionnaire of the European Organization for Research and Treatment of Cancer; *EORTC QLQ-FA13* 13-item fatigue questionnaire of the European Organization for Research and Treatment of Cancer; *FCQI* Freiburg Questionnaire of Coping with Illness

In the case of nonresponse to the follow-up questionnaires at 3 and 12 months after discharge, participants receive three additional reminders. The first reminder consists of a letter and is sent one week after the initial follow-up questionnaire; the second reminder includes the questionnaire again and is sent three weeks after the initial follow-up questionnaire; the third reminder also includes the questionnaire again and is sent six weeks after the initial follow-up questionnaire.

### Main study outcome

The primary outcome of the study is the role functioning scale of the 30-item quality-of-life questionnaire of the European Organization for Research and Treatment of Cancer (EORTC QLQ-30). The role functioning scale comprises two items that relate to a) work and other daily activities and b) hobbies and leisure time activities. Response categories are “not at all,” “a little,” “quite a bit,” and “very much.” Single-item scores will be averaged and transformed to a score from 0 to 100 points. Higher scores represent better functioning [56].

### Secondary study outcome

Secondary outcome measures are additional scales of the EORTC QLQ-30. These scales measure physical functioning, emotional functioning, social functioning, pain, and global health [56, 57]. Additionally, we use the 13-item fatigue module (EORTC QLQ-FA13) [58]. All scales range from 0 to 100 points. Higher scores indicate a better health-related quality of life.

Self-rated work ability is measured with the Work Ability Score. Ten points represent the best work ability ever achieved; 0 points indicate total work disability [59].

Disease coping is measured with three scales of the Freiburg Questionnaire of Coping with Illness (FCQI; depressive coping, active coping, distraction, and self-encouragement) with 5-point scaled items [60]. Raw item scores will be added up to a sum score for each scale. These will be used to calculate a mean scale score, resulting in values ranging from 1 to 5 points [60].

Participation in working life is measured with an assessment of the current employment status, current sick leave status, and disability days during the last 3 months [61, 62]. The assessment after 12 months also includes a question regarding the date of initial return to work.

The realization of work-related goals and therapies during rehabilitation is assessed with a slightly modified version of a previously used set of items from a study that investigated the implementation of the WMR guidelines [63]. Participants report on 12 dichotomized items whether they received WMR contents throughout their rehabilitation program. Scores are aggregated to a total score ranging from 0 to 12 points. This score reflects the implementation of the work-related therapies. Additionally, 6 items assess the perceived diagnostic and therapeutic focus on issues of return to work and work ability, e.g. the experience of a consistent RTW strategy. These items are 5-point scaled. Scores will be summed to a total score ranging from 0 to 24 points. Finally, the subjective work-related benefit from participating in the rehabilitation program is assessed with 8 items using 5-point scales. Scores are aggregated to a total score ranging from 0 to 32 points.

Treatment satisfaction is assessed using the German version of the Client Satisfaction Questionnaire (CSQ-8) [64]. This questionnaire uses 8 items to assess various aspects of the patient’s satisfaction with the treatment. Items are 4-point scaled. The sum score ranges from 8 to 32 points.

### Supplemental data

The sociodemographic data that are assessed include age, sex, employment status, native language, partnership, number of children, educational level, and job qualification. Additionally, a discrete choice experiment is attached to the baseline questionnaire to assess patients’ treatment preferences [65].

### Data management

Questionnaires will be scanned and verified by an electronic data capture system and exported to statistical software packages for further analysis. Scanning and verifying is done by trained research assistants. Research assistants check electronically processed data item by item and compare imported data with the original questionnaire data.

If available, standard syntaxes are used to calculate scales (e.g. symptom scales of the EORTC QLQ-30) for further analyses. Each data set is checked for errors or inconsistencies before merging them with data from the other data sources or time points via the assigned study number to create a comprehensive data set. Data access is limited to the authors and the research assistants of the research team at the University of Lübeck.

### Statistical analysis

Treatment effects are tested using regression analyses. In the case of binary study outcomes, logistic regression will be used (e.g. effects on employment status or sick leave at follow-up). For analysis of time to return to work, we will use proportional hazard models. Baseline parameters will be considered as covariates [66]. Due to the cluster-randomization, the induced correlation of error terms for patients with the same arrival week will be taken into account by a random effect [67–69]. Rehabilitation centers will be considered using a fixed effect.

To calculate a SMD of the between-group difference, the unstandardized regression coefficient estimate of the treatment effect will be used [70]. This estimate will be standardized using the pooled standard deviation of the observed follow-up scores [71]. In the case of binary study outcomes, estimated odds ratios will be transformed into SMDs using a logit-transformation [71, 72]. SMDs will be interpreted as suggested by Cohen [73]: small effect: SMD ≥0.2, medium effect: SMD ≥0.5, large effect: SMD ≥0.8.

Furthermore, to test whether the treatment effect on the primary outcome is moderated by other variables we will include two-way interaction terms in the regression model. Potential moderators that will be tested are the remaining EORTC QLQ-30 scales, fatigue, the Work Ability Score, disease coping, and the type of rehabilitation (rehabilitation following primary cancer treatment vs. follow-up rehabilitation) as well as sociodemographic variables. Continuous moderators will be standardized using a z-transformation. The parameter estimate of the interaction between the z-standardized continuous score and the treatment indicator then represents the additional treatment effects when increasing the potential moderator by about one standard deviation [74–76]. Patients will be included in our analyses if they have completed the corresponding follow-up questionnaire and will be analyzed as randomized, i.e. as intended to treat [77]. Differences will be regarded as significant if the two-sided p-value of a test is less than 0.05.

Missing observations are excluded from the primary analysis. Supplementary analyses will impute missing observation by using the last observation carried forward method, in which the last observed value is used in place of the missing endpoint. No stopping guidelines are applied and no adverse health events are expected.

## Discussion

Due to the increasing survival rates of cancer patients, the specific role of RTW in coping with the disease, and the higher risk of being excluded from the labor market, a clear need for interventions that support work-related outcomes can be identified. The study will provide highly ranked evidence for or against the effectiveness of WMR in cancer patients when compared to conventional MR. Positive results in favor of WMR might be expected considering the already existing body of evidence regarding WMR for other health conditions [40–45]. The current study addresses the shortcomings of previous studies by applying a randomized controlled study design and ensuring implementation of the WMR programs according to current guidelines in all participating centers. A positive evaluation of WMR in cancer patients might support the dissemination of WMR programs in German cancer rehabilitation.

## Trial status

The implementation of the work-related medical rehabilitation program in all participating rehabilitation centers is finished. Patient recruitment started in June 2015 and is ongoing. For regular updates, please check the trial register entry. Items from the World Health Organization trial registration data set are presented in Additional file 1.

## Abbreviations

CSQ, Client Satisfaction Questionnaire; EORTC QLQ-30, 30-item quality-of-life questionnaire of the European Organization for Research and Treatment of Cancer; EORTC QLQ-FA13, 13-item fatigue questionnaire of the European Organization for Research and Treatment of Cancer; FCQI, Freiburg Questionnaire of Coping with Illness; GPI, German Pension Insurance; ICD, International Statistical Classification of Diseases and Related Health Problems; MR, medical rehabilitation; RTW, return to work; SIBAR, Screening Instrument Work and Occupation (German: Screening-Instrument Beruf und Arbeit in der Rehabilitation); SMD, standardized mean difference; SPIRIT, Standard Protocol Items: Recommendations for Interventional Trials; WMR, work-related medical rehabilitation

## Additional files

Additional file 1: Table S1. World Health Organization trial registration data. (DOCX 19 kb) Additional file 2: SPIRIT 2013 Checklist. (DOC 123 kb) Additional file 3: Translated study information for rehabilitation patients. (DOCX 53 kb)
