# Supplementary material for: Effectiveness of work-related medical rehabilitation in cancer patients: study protocol of a cluster-randomized multicenter trial
Source: BMC Cancer. 2016 Jul 27;16:544. doi: 10.1186/s12885-016-2563-z (PMC4964285; doi:10.1186/s12885-016-2563-z)
Supplement: Additional file 1: Table S1. — World Health Organization trial registration data. (DOCX 19 kb) [file 12885_2016_2563_MOESM1_ESM.docx]

**Table S1** World Health Organization trial registration data

| Data category | Information |
| --- | --- |
| Primary registry and trial identifying number | German Clinical Trials Register  DRKS00007770 |
| Date of registration in primary registry | 13 May 2015 |
| Secondary identifying numbers | 14-289, U1111-1145-4678 |
| Primary sponsor | Federal German Pension Insurance |
| Contact for public queries | Name: Julian Wienert, PhD  Address: Ratzeburger Allee 160, 23562 Lübeck, Germany  Telephone: +494515005878  Email: julian.wienert@uksh.de  Affiliation: University of Lübeck, Institute for Social Medicine and Epidemiology, Section Rehabilitation and Work |
| Contact for scientific queries | Name: Julian Wienert, PhD  Address: Ratzeburger Allee 160, 23562 Lübeck, Germany  Telephone: +494515005878  Email: julian.wienert@uksh.de  Affiliation: University of Lübeck, Institute for Social Medicine and Epidemiology, Section Rehabilitation and Work |
| Public title | Effectiveness of work-related medical rehabilitation in cancer patients: a cluster randomized multicenter trial |
| Scientific title | Effectiveness of work-related medical rehabilitation in cancer patients: a cluster randomized multicenter trial - WMR-C |

| Countries of recruitment | Germany |
| --- | --- |
| Health condition(s) or problem(s) studied | C00-D48 - Neoplasms |
| Intervention(s) | Intervention 1: Participants receive the standard rehabilitation program and the following treatments which focus specifically on return to work: social counseling, work-related psychological group treatments and work-related functional capacity training.  The social counseling (min. 90 minutes, 30 minutes of these in a one-to-one setting) considers the following themes: occupational situation and perspective, sickness benefits, rights for severely disabled people, employer disability management, graded return-to-work, aids/services for participation in work life, and chances and risks of disability pensions.  The work-related psychological group treatments (min. 240 minutes) have the following contents: coping with work-related stress, work-related social competences, and barriers and supportive factors for return to work.  The work-related functional capacity training (min. 390 minutes, 30 minutes of these for initial individual functional capacity evaluation) includes: training of typical work-related movements, cognitive training, compensatory exercises, ergonomics.  The work-related treatments are introduced and reasoned by a physician in an initial group session. |
|  | Intervention 2: Participants receive the usual medical rehabilitation program in accordance to the guidelines of the German Pension Insurance Agency |

| Key inclusion and exclusion criteria | Age minimum: 18 years Age maximum: 60 years Gender: Both, male and female |
| --- | --- |
|  | Inclusion criteria: poor work ability (positive rating at one of the three scales of the SIBAR (Screening-Instrument Beruf und Arbeit, Bürger und Deck 2009); Karnofsky Performance Status Scale = 70% (Karnofsky et al. 1948); positive social-medical prognosis (employability of min. 3 hours/day in no later than 6 months) |
|  | Exclusion criteria: None |
| Study type | Interventional |
|  | Allocation: Randomized controlled trial;. Masking: Open (masking not used). Control: Active control (effective treament of control group). Assignment: Parallel. |
|  | Primary purpose: Treatment |
| Date of first enrolment | 01/06/2015 |
| Target sample size | 504 |
| Recruitment status | Recruiting |
| Primary outcome(s) | Primary outcome will be assessed at the beginning of cancer rehabilitation (T1), as well as 3 months (T3) and 12 months (T4) after the end of cancer rehabilitation with a questionnaire: role functioning scale (T1, T3, T4, EORTC QLQ-C30, Aaronson et al. 1993). |

| Key secondary outcomes | Secondary outcomes will be assessed at the beginning of cancer rehabilitation (T1), at the end of cancer rehabilitation (T2), as well as 3 months (T3) and 12 months (T4) after the end of cancer rehabilitation with a questionnaire. Secondary outcomes are physical functioning (T1, T2, T3, T4, EORTC QLQ-C30, Aaronson et al. 1993), emotional functioning (T1, T2, T3, T4, EORTC QLQ-C30, Aaronson et al. 1993), social functioning (T1, T3, T4, EORTC QLQ-C30, Aaronson et al. 1993), pain (T1, T2, T3, T4, EORTC QLQ-C30, Aaronson et al. 1993), global health (T1, T2, T3, T4, EORTC QLQ-C30, Aaronson et al. 1993), fatigue (T1, T2, T3, T4, EORTC FA-13, Weis et al. 2013); work ability score (T1, T2, T3, T4, Ilmarinen 2007), disease coping (T1, T2, T3, T4, questionnaire on disease coping, Muthny 1989), employment status (T1, T3, T4), disability days (T1, T3, T4, von Korff et al. 1992), time of return to work (T3, T4); implementation of work-related therapies (T2, Bethge et al. 2014), consistency of work-related rehabilitation strategy (T2, Bethge et al. 2014), benefit from work-related therapies (T2, Bethge et al. 2014), treatment satisfaction (T2, Schmidt et al. 1989), received therapeutic treatments (T2, discharge letter), social-medical capacity evaluation (T2; discharge letter). |
| --- | --- |
