# Supplementary material for: Effectiveness of work-related medical rehabilitation in cancer patients: study protocol of a cluster-randomized multicenter trial
Source: BMC Cancer. 2016 Jul 27;16:544. doi: 10.1186/s12885-016-2563-z (PMC4964285; doi:10.1186/s12885-016-2563-z)
Supplement: Additional file 3: — Translated study information for rehabilitation patients. (DOCX 53 kb) [file 12885_2016_2563_MOESM3_ESM.docx]

| 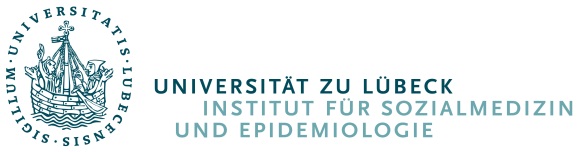 |
| --- |

**Study Information**

*Dear patient,*

This letter informs you about the study:

***„Effectiveness of work-related medical rehabilitation in cancer patients: a cluster randomized multicenter trial“.***

We – the researchers – would like to ask you to participate in our study. In the present patient information you can find our contact details and details about your contact person at your rehabilitation center which will help us to realize the study (paragraph 1). Paragraph 2 explains the major aim of our study. The procedure of our study and your potential contribution are outlined in paragraph 3. As data protection is as important for us, as it is for you, we describe in paragraph 4 and 5 from where and how we get the research data and how we handle them.

Based on this patient information you can make an informed decision if you would like to participate in our study or not. You can keep this patient information for yourself.

**1) Who are the responsible contact persons for the study?**

The study is led by Prof. Dr. Matthias Bethge from the University of Lübeck (Institute for Social Medicine and Epidemiology, Section for Rehabilitation and Work, Ratzeburger Allee 160, 23538 Lübeck).

In case you have any questions regarding the study you can contact Dr. Julian Wienert from the University of Lübeck (Tel. 0451 500-5878, E-Mail: julian.wienert@uksh.de) who is entrusted with conducting the study.

Your direct contact person in your rehabilitation center is: *[Name of the study nurse and room]*.

Our study is funded by the Federal German Pension Insurance.

**2) What is the aim of our study?**

Two treatment programs currently are used in cancer rehabilitation: conventional medical rehabilitation and work-related medical rehabilitation. Still, it is unclear if one of the two achieves better treatment outcomes.

Therefore, our study focuses on rehabilitation patients with cancer who receive a conventional medical rehabilitation or a work-related medical rehabilitation and compares their treatment outcomes.

**3) How is the study performed? What may be your contribution?**

As part of our study all patients, who arrive this week, are either assigned to conventional medical rehabilitation or to work-related medical rehabilitation by chance. To test whether one of the two programs achieves better treatment outcomes we provide ***paper-and-pencil questionnaires*** at four time points: at the beginning and at discharge of rehabilitation as well as 3 and 12 month after the end of rehabilitation. The questionnaires comprise 3 to 6 pages and deal with your health, your functional status, and your treatment satisfaction. If you wish, you can request the questionnaires prior to consenting in participation from your local contact person at the rehabilitation center. It takes approximately 30 minutes to fill in one questionnaire. Additionally, we would like assess data from your ***medical discharge letter***. These data comprise information about your therapeutic treatments during your rehabilitation, details from your treatment diagnosis, the discharge mode, as well as your work capacity and recommendations for follow-up treatment.

**4) How are my data handled? Who receives the names of participants?**

When giving informed consent to participate in our study, your name and address will be listed by your rehabilitation center and a study number will be assigned. This study number will be entered in a field on your questionnaires and will also be added to the data from your medical discharge letter. Thus, we get no information on your name and address. This procedure is called pseudonymization and ensures that research data and confidential data cannot be linked. We can merely use the number to connect the data from both data sources (i.e. the data from your questionnaires and your medical discharge letter). This is very important for us, as we are interested in investigating treatment trajectories and outcomes. The study list with your personal data and the study number will be destructed by your rehabilitation center at the end of the project (12/31/2017) to ensure that names and addresses of study participants are no longer available.

We, the researchers, receive a copy of this study list only for mailing the questionnaires 3 and 12 months after discharge. Use of these data is not allowed for any other purpose but sending out the questionnaires. This study list will be secured with a password, stored separately from the collected research data, and erased after sending out the last questionnaires.

Following this procedure we ensure that no one can link the research data with your name. However, during the time of data collection the research data are treated as “personal data” within the meaning of the data privacy act. We are only allowed to collect these data with your explicit, voluntary, and written consent. Additionally, all researchers are obliged to treat your data confidential.

**5) Does my pension insurance receive any data?**

The pension insurance receives no information about your participation or non-participation. The researchers only inform the pension insurance about the realization of the study with anonymous results.

**Voluntariness**

Your participation is voluntary. **Your data will only be used when you sign the informed consent form.** In case you do not wish to participate, you do not have to complete the informed consent form. You do not have to justify your decision.

You will not experience any negative consequences neither from participation nor from non-participation. You can withdraw your consent at any time without naming any reason. In this case, please inform your contact person from your rehabilitation center listed above. We will then delete your name from the study list as well as all collected research data, if information can still be linked.

**Request upon participation**

If you have read the above, understood its content and wish to participate in the study, we would like to ask you to sign the informed consent form. There are two copies of this form. One of the forms is for the rehabilitation center. The second form is for you. Please keep this information sheet so that you can remember what you have consented for.

We would be happy if you are interested in participating in our study. The more persons participate the more informative are the results.

**Prof. Dr. Matthias Bethge**

**Principle Investigator**
